# Bevacizumab withdrawal–associated cortical hyperperfusion in recurrent high-grade astrocytoma: An underrecognized MRI pitfall

**DOI:** 10.1016/j.radcr.2026.03.027

**Published:** 2026-04-15

**Authors:** Alice Coelho, Zhijian Chen, Charif Sidani, Kevin J Abrams, Leonardo Furtado Freitas

**Affiliations:** a American University of Antigua, Saint John's, Antigua and Barbuda; b Division of Neuro-oncology, Baptist Health South Florida, Miami Cancer Institute, Miami, FL, USA; c Herbert Wertheim College of Medicine, Florida International University (FIU), Miami, FL, USA; d Baptist Health South Florida, Miami, FL, USA; e Division of Clinical Neuroradiology, Department of Radiology, Radiology Associates of South Florida (RASF), Miami, FL, USA

**Keywords:** Bevacizumab, Glioma, Perfusion MRI, Hyperperfusion, Anti-angiogenic therapy, Treatment-related imaging findings

## Abstract

Bevacizumab is widely used in recurrent high-grade gliomas due to its anti-angiogenic effects mediated through vascular endothelial growth factor (VEGF) inhibition. While radiological pseudo response during treatment is well recognized, cerebral hemodynamic alterations following treatment discontinuation are poorly characterized. We report a 50-year-old male with recurrent IDH-mutant WHO grade 4 astrocytoma who developed focal cortical hyperperfusion on perfusion MRI after cessation of long-term Bevacizumab therapy. The finding occurred in the absence of diffusion restriction, seizure activity, systemic hypertension, stroke episode or imaging evidence of tumor recurrence. The imaging pattern is most consistent with a transient vascular autoregulatory rebound phenomenon related to vascular endothelial growth factor pathway re-equilibration after anti-angiogenic withdrawal.

## Introduction

Bevacizumab, a monoclonal antibody targeting VEGF-A, exerts its therapeutic effects by inhibiting angiogenesis and modulating tumor-associated vasculature. In high-grade gliomas, vascular endothelial growth factor (VEGF) blockade reduces vascular permeability, restores elements of blood–brain barrier integrity, and decreases vasogenic edema, frequently producing radiographic improvement referred to as pseudo response. However, VEGF inhibition also induces significant endothelial and microvascular alterations, including reduced nitric oxide bioavailability, increased vascular tone, and microvascular rarefaction. Although the vascular effects of Bevacizumab during active treatment have been extensively described, less is known about the cerebrovascular consequences of drug discontinuation. We describe a case of isolated cortical hyperperfusion following suspension of prolonged Bevacizumab therapy in a patient with recurrent astrocytoma, emphasizing its differentiation from tumor progression.

### Case presentation

A 50-year-old male was diagnosed in September 2021 with a left frontal WHO grade 4 astrocytoma, IDH-mutant and MGMT promoter methylated. He underwent gross total resection followed by radiotherapy and adjuvant chemotherapy with lomustine and Temozolomide according to the Herlinger protocol, completing 6 cycles in July 2022. In February 2023, follow-up brain MRI demonstrated increased relative cerebral blood volume (rCBV - dynamic susceptibility contrast (DSC) MRI with T2*-weighted sequence). Multimodal imaging, including MRI with spectroscopy and PET/MRI, revealed a slight increase in the T1 post-contrast enhancing lesion, elevated rCBV, and increased FDG uptake at the site of enhancement, findings suspicious for disease progression.

**Fig. 1:**
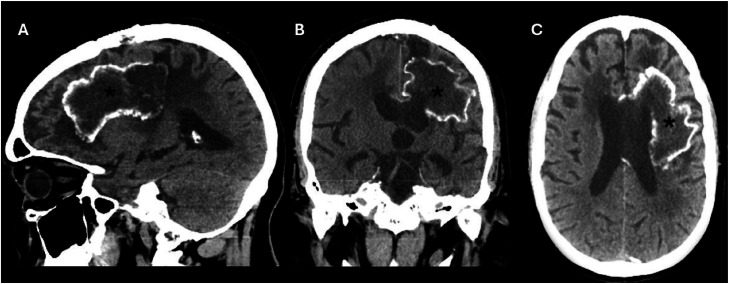
Non-contrast head computed tomography demonstrating sagittal (A), coronal (B), and axial (C) reformatted images. A large lobulated lesion with peripheral calcifications is identified in the anterior aspect of the left centrum semiovale/corona radiata (asterisks), showing central hypoattenuation suggestive of treatment-related changes (coagulative necrosis induced by antiangiogenic therapy). Additional cortical hypoattenuation is noted in the left frontal lobe, consistent with sequelae.

**Fig. 2:**
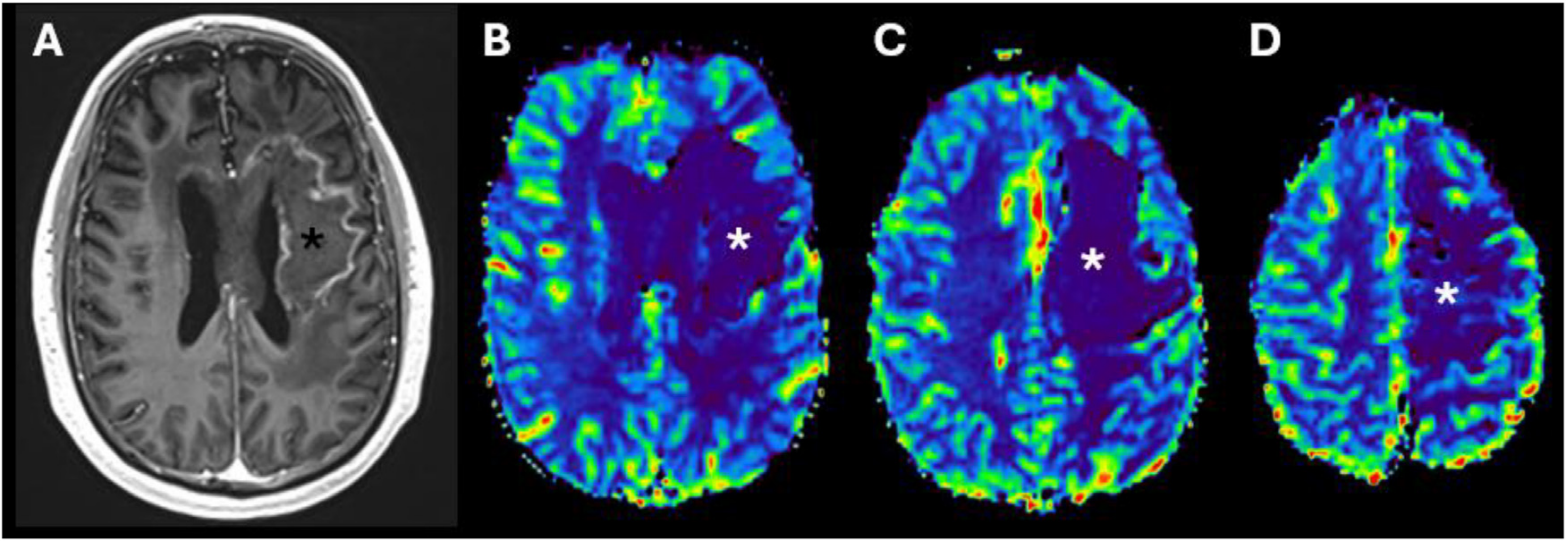
Brain magnetic resonance imaging (MRI) performed while the patient was still receiving antiangiogenic therapy, including axial post-gadolinium T1-weighted MPRAGE volumetric sequence (A) and relative cerebral blood volume (rCBV) T2*-weighted perfusion maps (B–D). The lesion corresponding to treatment-related necrosis (asterisks) demonstrates peripheral contrast enhancement and marked hypoperfusion (low rCBV). Careful evaluation of the perfusion pattern in the left frontal lobe also reveals mild hypoperfusion.

The patient was subsequently enrolled in a Phase I clinical trial evaluating D2C7-IT in combination with an Fc-engineered anti-CD40 monoclonal antibody administered intratumorally via convection-enhanced delivery, and the treatment began on June 15, 2023. Bevacizumab therapy at a dose of 7.5 mg/kg every 3 weeks was initiated on July 21, 2023. A second radiological progression was identified in December 2023, prompting treatment with metronomic temozolomide in combination with Bevacizumab. A third progression was documented in February 2024, after which the patient underwent re-irradiation completed in April 2024 with concurrent Bevacizumab. Combined therapy was continued thereafter, with 5 cycles of lomustine completed in November 2024. Bevacizumab monotherapy was maintained on a monthly schedule until December 9, 2025 (Fig. 1, Fig. 2), when therapy was suspended due to hospitalization.

### Imaging findings following bevacizumab discontinuation

**Fig. 3:**
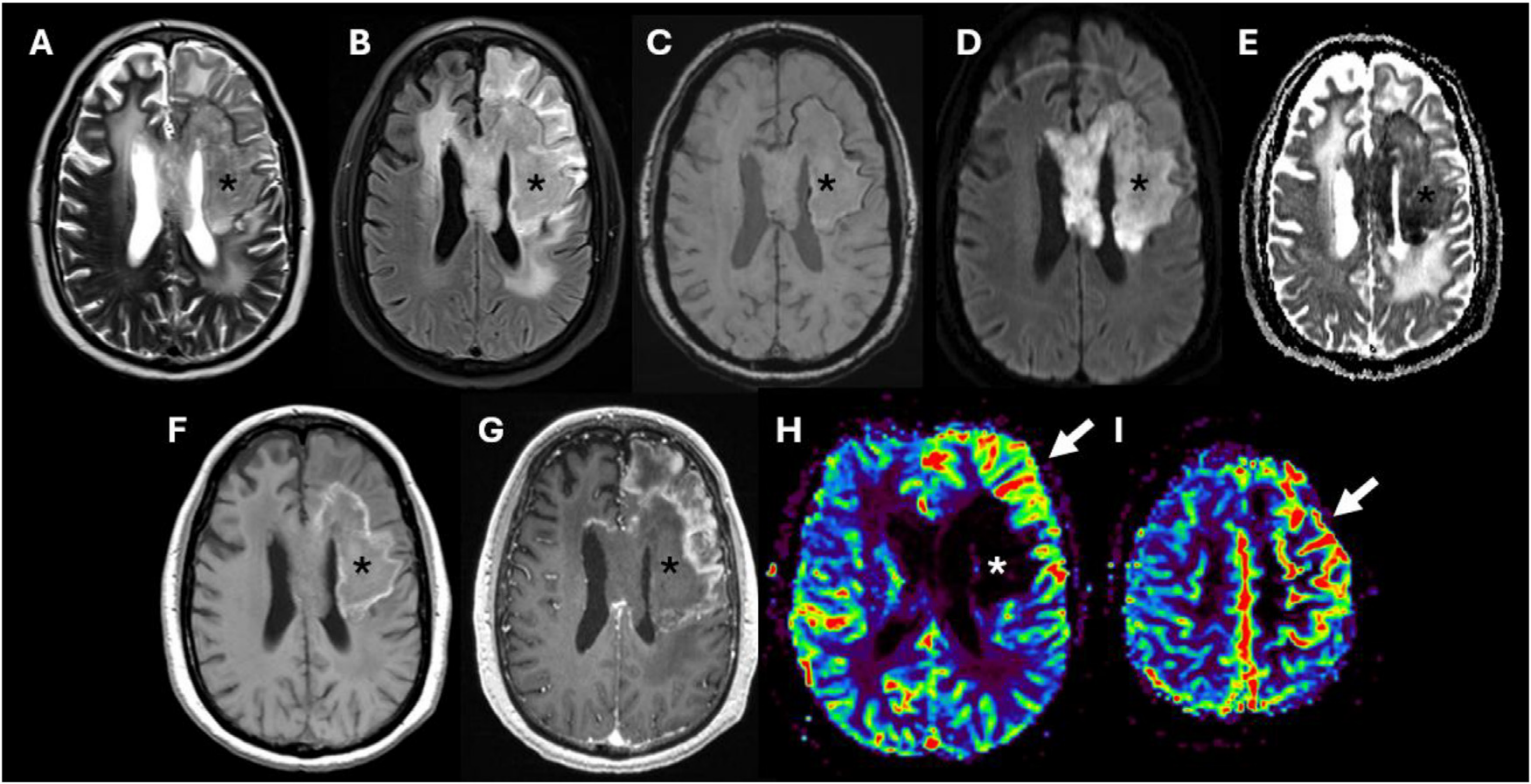
Two-month follow-up brain MRI demonstrating axial T2-weighted (A), FLAIR (B), susceptibility-weighted imaging (SWI) (C), diffusion-weighted imaging (DWI) (D), apparent diffusion coefficient (ADC) map (E), axial T1-weighted without contrast (F), axial post-contrast T1-weighted (G), and relative cerebral blood volume (rCBV) perfusion maps (H, I). At the time of imaging, anti-angiogenic therapy had been suspended for 2 months. Similar features of treatment-related necrosis (asterisks) with marked diffusion restriction on DWI with corresponding low ADC values. Additionally, new gyriform enhancement (G, white arrows) is identified in the left frontal lobe – superior, middle and pre central gyri -, associated with marked hyperperfusion on perfusion imaging (white arrows) and no diffusion restriction.

Two months after cessation of bevacizumab, follow-up perfusion MRI (Fig. 3) demonstrated left frontal cortical hyperperfusion characterized by increased cerebral blood volume within the ipsilateral cortex, with associated gyriform enhancement, adjacent to the radiation field and deep enhancing area. This finding did not demonstrate restricted diffusion and the apparent diffusion coefficient map was unremarkable. There was no infiltrative signal abnormality suggestive of tumor progression, and no significant mass effect. The patient did not exhibit acute neurological decline or seizure activity, with a negative EEG.

The imaging pattern was therefore considered inconsistent with tumor progression, acute ischemia, SMART syndrome, or posterior reversible encephalopathy syndrome.

## Discussion

This case most plausibly represents a transient vascular autoregulatory phenomenon occurring after Bevacizumab withdrawal. Under physiological conditions, VEGF signaling promotes endothelial nitric oxide synthase activation and nitric oxide production, maintaining vasodilatory tone and endothelial homeostasis. Chronic VEGF blockade with Bevacizumab reduces nitric oxide bioavailability, increases vascular tone, and induces microvascular rarefaction. These changes contribute to vascular normalization and decreased permeability during treatment, accounting for reduced enhancement and edema frequently observed on MRI.

Following discontinuation of prolonged VEGF inhibition, reactivation of VEGF-mediated endothelial signaling may result in transient dysregulation of cerebrovascular tone. The sudden removal of sustained anti-angiogenic pressure may permit reactive vasodilation and localized hyperemia, manifesting as increased regional cerebral blood volume and blood-brain barrier disfunction on perfusion imaging.

Other causes of cortical hyperperfusion were considered. Post-ictal hyperperfusion may produce similar perfusion abnormalities; however, there was no clinical history of seizure activity and no associated diffusion restriction or transient cortical swelling. SMART syndrome may also present with cortical enhancement and perfusion abnormalities, but typically occurs in patients with prior cranial adiation and characteristic migraine-like episodes, which were absent in this case. In this clinical and imaging context, the findings are most consistent with vascular re-equilibration following withdrawal of VEGF inhibition rather than neoplastic progression.

Recognition of this potential imaging pitfall is essential in heavily pretreated glioma patients, in whom perfusion abnormalities may otherwise be misinterpreted as tumor recurrence. Careful correlation with diffusion imaging, enhancement characteristics, clinical status, and longitudinal follow-up is critical to avoid premature therapeutic escalation.

## Conclusion

Focal cortical hyperperfusion following cessation of bevacizumab may reflect a transient autoregulatory rebound phenomenon potentially related to restoration of VEGF-dependent endothelial signaling. Awareness of this imaging pattern may be important for the interpretation of post-treatment perfusion MRI in patients with high-grade glioma and could help avoid misclassification of vascular phenomena as tumor progression. Further investigation is needed to better define the incidence and clinical significance of cerebral hyperperfusion after withdrawal of anti-angiogenic therapy.

To our knowledge, cortical hyperperfusion following bevacizumab discontinuation has not been previously described as a distinct imaging finding in the literature.

Although other perfusion-related and vascular autoregulatory disturbances associated with anti-VEGF therapy have been reported—such as hypertension [1], cerebral infarction [2], and posterior reversible encephalopathy syndrome [3]—post-withdrawal hyperperfusion has not been previously characterized. While the mechanism of VEGF pathway re-equilibration remains hypothetical, recognition of this imaging pattern may broaden the spectrum of cerebrovascular alterations potentially associated with anti-angiogenic therapy and highlights the dynamic effects of VEGF pathway modulation in the central nervous system.

## Author contributions

Alice Coelho: Data collection and manuscript drafting. Zhijian Chen: Clinical management and manuscript review. Charif Sidani: : Clinical management and manuscript review. Kevin J Abrams: Imaging interpretation and manuscript review. Leonardo Furtado Freitas: Case conception, imaging interpretation, manuscript drafting, and supervision.

## Patient consent

Written informed consent for the publication of this case report was obtained from the patient.
